# Very High Negative Concordance Rate of RT-PCR for SARS-CoV-2 in Nasopharyngeal Swab and Tracheo-Bronchial Aspirate in Children

**DOI:** 10.3389/fped.2022.866111

**Published:** 2022-05-25

**Authors:** Anna Camporesi, Annalisa De Silvestri, Veronica Diotto, Stefania Ferrario, Laura Eccher, Alessandra De Ferrari, Francesco Messina, Gloria Pelizzo, Davide Mileto, Valeria Calcaterra, Danilo Buonsenso

**Affiliations:** ^1^Department of Pediatric Anesthesia and Intensive Care, “Vittore Buzzi” Children's Hospital, Milan, Italy; ^2^Clinical Epidemiology and Biometry Unit, Fondazione IRCCS Policlinico San Matteo, Pavia, Italy; ^3^Pediatric Ear, Nose and Throat Unit, “Vittore Buzzi” Children's Hospital, Milan, Italy; ^4^Department of Pediatric Surgery, “V. Buzzi” Children's Hospital, University of Milan, Milan, Italy; ^5^Department of Biomedical and Clinical Sciences, “Luigi Sacco” University Hospital, Milan, Italy; ^6^Clinical Microbiology, Virology, and Bio-Emergence Diagnosis, ASST Fatebenefratelli-Sacco, Department of Biomedical and Clinical Sciences, University of Milan, Milan, Italy; ^7^Department of Pediatrics, “Vittore Buzzi” Children's Hospital, Milan, Italy; ^8^Department of Woman and Child Health and Public Health, Fondazione Policlinico Universitario “A. Gemelli”, Rome, Italy

**Keywords:** SARS-CoV-2, naso-pharyngeal swab, tracheo-bronchial aspirate, children, RT-PCR-Real-Time PCR

## Abstract

Reliable testing methods for severe acute respiratory syndrome coronavirus 2 (SARS-CoV-2) in children are essential to allow normal activities. Diagnosis of SARS-CoV-2 infection is currently based on real-time reverse transcriptase-polymerase chain reaction (RT-PCR) performed on nasopharyngeal (NP) swabs; concerns have been raised regarding NP swab accuracy in children to detect the virus because of potential lack of cooperation of the patients or due to general uncertainties about concordance between high and low respiratory tract specimens in children. The aim of the study (IRB approval: ST/2020/405) is to prospectively compare RT-PCR results on NP and tracheo-bronchial aspirate (TA) in children admitted to the hospital for surgery or admitted to the Pediatric Intensive Care Unit (PICU) of a tertiary children hospital in Milano, Italy, during a peak of COVID-19 infections in the city. A total of 385 patients were enrolled in the study: 364 from surgical theater and 21 from PICU. Two patients (0.5%) tested positive on TA and were negative on NP; both cases occurred in November 2020, during a peak of infection in the city. Specificity of NP swab was.995 (95% CI: 0.980–0.999). Two patients with positive NP swabs tested negative on TA.

**Conclusion:**

Our study shows that the specificity of SARS-CoV-2 RT-PCR on TA swab, compared to results of SARS-CoV-2 RT-PCR on NP, was very high for negative cases in our pediatric cohort during a period of high epidemiological pressure.

## Introduction

While children have shown lower incidence and severity of COVID-19, they have often had restrictions placed on their activities because they are considered a potential reservoir for the disease and source of infection for the adult population (1).

Diagnosis of SARS-CoV-2 infection is currently based on RT-PCR performed on nasopharyngeal (NP) swabs (2). The same diagnostic method can also be applied to other specimens (sputum, tracheal aspirate, bronchoalveolar lavage, urines, feces, etc.) (3). However, concerns have been raised regarding NP swab accuracy in children to detect the virus because of the potential lack of cooperation of the patients (4) or due to general uncertainties about concordance between high and low respiratory tract specimens in children with viral and bacterial respiratory infections in previous studies (5). While alternative methods to collect suitable material for SARS-CoV-2 research in upper respiratory tract specimens, such as nasal and nasopharyngeal fluid (6), have been described, the current standard diagnostic method in the pediatric population remains NP swab.

This prospective study aims to compare RT-PCR results on NP and tracheo-bronchial aspirate (TA) in children.

## Methods

This is a prospective observational study conducted at a tertiary pediatric hospital in Milano, Italy (IRB approval: ST/2020/405) between 2 November 2020 and 2 June 2021 on children admitted to the hospital for surgery or admitted to the Pediatric Intensive Care Unit (PICU). The chosen period coincided with the peak of COVID-19 infections in the city (7). All consecutive patients meeting the inclusion criteria underwent NP and TA RT-PCR for SARS-CoV-2.

### Usual Testing Pathway for Patients Admitted to Hospital

All patients admitted to our hospital undergo an NP swab, together with the caregiver who will stay in the hospital with them; in the case of planned surgery, this is obtained 48 h before surgery, and if it gives a positive result for any of the two (patient and caregiver), surgery is postponed. In the time frame between NP swab and surgery, both patient and caregiver are officially quarantined. The maximal interval between evaluation with swab and surgery is 72 h; after this time, if surgery is for any reason delayed, the swab is to be repeated.

In case of urgent/emergent surgery, patients receive NP swabs upon hospital admission and are treated as suspected cases in both ward and Operatory Room (OR) until the result of the swab is available for both patient and caregiver. If one of the two is positive, the case is treated as positive throughout the hospital stay.

Patients admitted to the PICU are tested with an NP swab together with the caregiver; until the result of the swab, patients are treated as positive cases even if admitted for non-respiratory reasons. If they are intubated, a sample of TA is collected for SARS-CoV-2 RT-PCR too.

For both surgical and patients in PICU, if in case the TA was positive, then the patient was treated as positive, and NP was repeated according to local protocols^1^.

### RT-PCR

On available samples, molecular analyses are performed to detect SARS-CoV-2 RNA, using the automated Real-Time PCR ELITe InGenius^®^ system and the GeneFinder^TM^ COVID-19 Plus RealAmp Kit assay (ELITechGroup, France). The reaction mix is manually prepared, according to the instructions of the manufacturer and loaded into the system with other reagents, while RNA is extracted from 200 μl of sample and eluted in 100 μl; the final reaction volume consists of 5 μl of RNA plus 15 μl of reagents mix. The RT-PCR profile is set up as follows, according to the instructions of the manufacturer: 50°C for 20 min, 95°C for 5 min plus 45 cycles at 95°C for 15 s, and 58°C for 60s. Three targeted regions in the RNA-dependent RNA polymerase (RdRP), Nucleocapsid (N), and Envelope (E) genes were simultaneously amplified and tested. A cycle threshold value (Ct-value) fewer than 40 is defined as a positive test result according to the instructions of the manufacturer.

### Patient Population

Inclusion criteria are defined as follows:

- Age 0–18 years.
- Tracheal intubation due to surgery requiring general anesthesia or tracheal intubation as part of life support in PICU.
- Written consent of caregiver.

During the enrolment period, samples of TA were collected from the anesthesiologist/intensivist in charge of the case. In the surgical patients, TA was collected after induction of anesthesia and intubation; in the patients in PICU, it was collected right after intubation if this occurred in the PICU, or upon admission, if the patient was transferred already intubated from another hospital.

When dealing with sample collection, the highest level of personal protective equipment was mandatory for the person involved (sheltering facepiece (FFP) 3 mask or equivalent; visor; long-sleeved gown; and gloves). After collection, the samples were immediately sent to the laboratory for analysis.

### Statistical Analysis

Categorical data are expressed as count and percentage; specificity is reported along with binomial exact 95% confidence interval. Quantitative data are expressed as mean and standard deviation or median and interquartile range (25-75th centile). Data were analyzed with Stata v17.0 (StataCorp USA).

## Results

A total of 385 patients have been enrolled in the study: 364 from surgical theater and 21 from PICU. Among the surgical patients' group, 213 (58.45%) were scheduled for elective procedures and 151 (41.55%) for urgent surgery. The mean age was 7.30 ± 4.89 years. No patient showed COVID-19-related symptoms.

Among PICU patients, 14 were intubated due to respiratory failure, 5 due to neurologic events, and 2 due to trauma/burns. The mean age was 5.85 ± 4.88 years.

Of the surgical group, 22 samples were insufficient for testing, leaving 342 adequate samples for study.

The total number of adequate samples for testing was therefore 363:342 from surgical theater and 21 from PICU.

In total, four patients tested positive for SARS-CoV-2 during the study (Table 1).

**Table 1:** Results of NP and TA samples in the studied cohort.

	**TA +, N**	**TA -, N**	**Total, N**
NP+, N	0	2	2
NP-, N	2	359	361
Total, N	2	361	363

*NP, naso-pharyngeal swab; TA, tracheo-bronchial aspirate; N, number*.

Two patients (0.5%), one in the surgical elective patients and one in the PICU group, tested positive on TA while being negative on NP; both cases occurred in November 2020.

Two urgent surgical patients, whose preoperative NP swab was positive, tested negative at TA.

Specificity of TA was 0.995 (95% CI: 0.980–0.999).

## Discussion

The NP swab is currently considered the “Gold Standard” for SARS-CoV-2 detection (8, 9). NP swab can however give false negative results, sometimes also related to suboptimal sample collection, especially in children (10). We decided to analyze and compare the results of both NP swab and TA in children who required intubation for surgical procedures or life support. Our cohort consisted mostly of healthy children, who do have a normal community life, who do not show COVID symptoms, and who had proven negative on the pre-operatory swab (and whose caregiver had tested negative too), but in a geographical and chronological setting characterized by high levels of virus circulation.

This is, to our knowledge, the first study analyzing concordance of results of SARS-CoV-2 RT-PCR in NP swabs and TA in children. Our results show that the specificity of TA was high in our cohort for the negative patients.

We cannot draw the same conclusion for the positive patients, where half of the cases were detected by NP and half cases by TA. It can be postulated that these results depend on a sample size problem, that is, the very low rate of positive patients that were enrolled in our cohort.

Overall, four patients in our study had discordant results. The negativity of RT-PCR on TA in the two patients whose NP swab was positive can be explained by a longer persistence of the virus in the upper respiratory tract compared to the lower respiratory tract. RT-PCR positivity could be due to persistent infection as well as the presence of non-transmissible virus fragments (11). We, unfortunately, do not have the relative viral cultures to check this possibility; it was not part of our protocol and the data collection was implemented during a period of high COVID-2019 circulation with relative resource limitation in all hospitals.

Another explanation can be that children usually mount a robust innate immune response within the upper airways that can limit the spread of the virus to the lower respiratory tract, as recently demonstrated in a study involving adult and pediatric patients (12).

Conversely, two patients had negative NP and positive TA tests. The discordance of upper and lower respiratory samples has been previously documented. Specifically, a recent study evaluated differences between swab results in the trachea and in the nasopharynx in 25 totally laryngectomized subjects, showing that results were overall divergent and no statistically significant correlations emerged between results of the tests performed in the two sites, suggesting that both tracheal and nasopharyngeal swabs are recommended in these kinds of patients, to obtain a reliable test and to avoid false negatives (13).

Our study has some limitations to address. First, the low number of positive samples did not allow us to calculate the sensitivity of the tests. Second, we did include only a small number of children with acute lower respiratory tract infections, therefore, our findings cannot be translated to these type of patients. Third, we neither have the viral load of the discordant samples or their ability to grow in culture nor the Cycle-Thresholds Values of RT-PCR which limits our ability to speculate if positive NP swabs with negative TA represent viral traces due to older infection.

In conclusion, our study showed that the specificity of SARS-CoV-2 RT-PCR on TA, compared to results of SARS-CoV-2 RT-PCR on NP, which is actually considered the gold standard, was very high for negative patients in our pediatric cohort, even during a period of high epidemiological pressure.

## Data Availability Statement

The raw data supporting the conclusions of this article will be made available by the authors, without undue reservation.

## Ethics Statement

The studies involving human participants were reviewed and approved by Comitato Etico Milano Area 1. Written informed consent to participate in this study was provided by the participants' legal guardian/next of kin.

## Author Contributions

AC designed the study and wrote the first draft of the manuscript. Material preparation, data collection and analysis were performed by AC, VD, SF, ADS, GP, DB, FM, ADF, LE, and DM. All authors commented on previous versions of the manuscript, read, and approved the final manuscript.

## Conflict of Interest

The authors declare that the research was conducted in the absence of any commercial or financial relationships that could be construed as a potential conflict of interest.

## Publisher's Note

All claims expressed in this article are solely those of the authors and do not necessarily represent those of their affiliated organizations, or those of the publisher, the editors and the reviewers. Any product that may be evaluated in this article, or claim that may be made by its manufacturer, is not guaranteed or endorsed by the publisher.

## Footnotes

^1^ http://www.salute.gwww.salute.gov.it
